# Compressive Strength Tests of Concrete Core Samples with the Addition of Recycled Aggregate

**DOI:** 10.3390/ma18112631

**Published:** 2025-06-04

**Authors:** Jacek Szpetulski, Grzegorz Sadowski, Bohdan Stawiski

**Affiliations:** 1 Faculty of Civil Engineering, Mechanics and Petrochemistry, Warsaw University of Technology, 17 Łukasiewicza St., 09-400 Płock, Poland; grzegorz.sadowski@pw.edu.pl; 2 Faculty of Environmental Engineering and Geodesy, Wroclaw University of Environmental and Life Sciences, 24 Grunwaldzki Sq., 50-363 Wrocław, Poland; bohdan.stawiski@upwr.edu.pl

**Keywords:** destructive test, compressive strength of concrete, core sample, recycled aggregate

## Abstract

Compressive strength tests of concrete using core samples are used to determine the strength of concrete elements in building structures. Due to ecology, the use of recycled aggregate in concrete is common. There are more and more concrete structures with recycled aggregate, in which the technical condition must be checked. It is difficult to find scientific studies concerning changes in compressive strength (using core samples of different sizes and using concrete with the addition of recycled aggregates) across the entire thickness of concrete elements. Therefore, studies of the compressive strength of core samples taken across the thickness (top layer, middle layer, bottom layer) of horizontally formed concrete elements with recycled aggregate and clean natural aggregate were conducted. The obtained test results allowed for the determination of the conversion coefficients that enable the compressive strength of the core samples (of different diameters: 59 mm, 74.5 mm, 114 mm, samples taken from different layers of a concrete element with a thickness of 260 mm) to be converted into the compressive strength of the core sample with a diameter of 94 mm and compared with a standard cubic sample with an edge length of 150 mm. The conversion coefficients can be used to determine the quality of the concrete produced or the technical condition of the building (mechanical damage, building reconstruction, building fire). The obtained results of the tests of the concrete samples, which had a compressive strength equal to 40 MPa and were prepared with the addition of recycled aggregate, indicate that there is a decrease of 17% in the strength value in the top layer of the concrete element when compared to its bottom layer. The concrete with a compressive strength of 20 MPa had a lower strength value of its top layer by 33% when compared to its bottom layer. Similar relationships were obtained for concrete with pure natural aggregate.

## 1. Introduction

Modern ecology aims to reduce the carbon footprint by recycling building materials. For this reason, recycled aggregate is used in concrete, which is a widely used material for building structures. In new buildings, concrete elements with recycled aggregate are increasingly used. Concrete, with the addition of recycled aggregate, has attracted the attention of researchers because it has positive economic and environmental impacts [1,2,3,4,5,6]. Concrete elements with aggregate from recycled building materials and pure natural aggregate may require compressive strength testing due to mechanical damage, building reconstruction, building fire, etc. Therefore, it is appropriate to establish conversion coefficients between the values of the compressive strength of core samples of different diameters taken from concrete elements.

Recycled materials added to concrete come from a variety of sources. The high-strength concrete containing iron ore rock waste is tested [7]. The concrete achieves a compressive strength of 61 MPa at 28 days, low porosity, and low carbonation depth. In this study, high-strength concrete was prepared using iron ore rock waste as coarse and fine aggregates in combination with cementitious materials based on solid waste (S95 slag and semi-dry desulfurization ash). In this publication [8] the conditions of preparation of ultramafic waste and steel slag were investigated. The tests were performed using orthogonal experiments. The test results show that the compressive strength has a positive exponential correlation with CO_2_ absorption by carbonated compacts. The authors proved that steel slag is the component hardening carbonized tailings–steel slag compacts. The researchers found that fibrous minerals in ultramafic tailings enforce the strength of the compacts. The publication [9] presents the production and characterization of carbonated lightweight aerated aggregates from waste paper fly ash—WPFA. Carbonated lightweight aggregates were made from WPFA (rich in CaO and with the addition of Barium and lead) by granulation with water in a high-intensity granulator. Physical, morphological, thermal, and mechanical characterization of lightweight aerated aggregates for use in mortars, concretes, or civil engineering structures was carried out. The results showed that Barium and lead were well immobilized in the solid matrix after carbonation.

The authors of the publications [4,10] showed that the use of recycled aggregates led to a decrease in the compressive strength of concrete and the fluidity of the concrete mix. It can be read in another publication [11] that recycled aggregates improved the compressive strength of concrete. The compressive strength of concrete with recycled aggregate decreases with the increase in the substitution rate of recycled aggregate [12]. The studies in publication [13] showed a significant effect of recycled aggregate in concrete on the penetration depth of pressurized water, permeability coefficient, and sorptivity.

The core samples are used to assess the compressive strength of concrete. The compressive strength of core samples depends on the following factors: the direction of drilling, the length-to-diameter ratio of the core, and the presence of reinforcement in concrete [14]. As per a study in publication [15], which studied different length-to-diameter ratios of drilled core samples, the change in length-to-diameter ratio affected the compressive strength results of small core samples more than the compressive strength results of 75 mm diameter core samples. Similar results were reported in publication [16], where the influence of diameter, concrete strength level, drilling direction, and length-to-diameter ratio on the compressive strength of concrete were confirmed. The results of the research [17] indicate that the compressive strength of the core of recycled aggregate concrete decreases with the increase in the aspect ratio by decreasing the core diameter and increasing the size of coarse aggregates in recycled concrete. The results of the studies in [18] indicate the influence of the shape and size of the sample on the obtained compressive strength in static tests.

The results in the publication [19] show that the cube strength is higher than the core strength for all grades of concrete under control and durability conditions. The cube test indicates a lower compressive strength than the Schmidt hammer test method at low-grade concrete under control conditions and greater compressive strength in the rest of the grades.

The authors of the publication [20] showed that the compressive strength of the cubic specimens was up to 19% higher than the compressive strength of the core sample of the control concrete with the addition of recycled aggregate. Moreover, the 100 mm diameter strength was about 10% lower than that of the 50 mm diameter core sample.

Publication [21] showed that the effect of reinforcement on core sample testing results decreased by decreasing the length-to-diameter ratio of the drilled core sample. Furthermore, they showed that the influence of a reinforcement ratio of up to 4% on the strength of core samples with a length-to-diameter ratio of 1 was insignificant.

The elastic modulus of concrete is an important parameter in building design. Empirical equations for the elastic modulus of concrete with natural aggregate NAC cannot be applied to concrete with recycled aggregate RAC. The elastic modulus of NAC is higher than that of RAC of the same strength, which, to some extent, hinders the widespread use of recycled aggregate. In article [22], an equation for the elastic modulus of RAC is presented based on a statistical analysis of 1383 concrete mixes from almost 160 publications. Such analysis enables designers to easily estimate the elastic modulus of RAC using known parameters at the design stage, such as compressive strength, replacement rate, and quality of recycled aggregate.

In publication [23], a new formula for estimating the elastic modulus of concrete containing recycled coarse aggregate was proposed. Artificial neural networks (ANN) and nonlinear regression were used for this purpose. The results indicated that this approach can be used to predict the elastic modulus of concrete. This proposal suggests better mix proportions for concrete containing natural and recycled coarse aggregates. The use of the simulation tool in the development of engineering projects allows for sustainable development.

The development of design guidelines for structural recycled aggregate concrete is hindered by the absence of a unified elastic modulus model focusing on the properties of diversely sourced coarse recycled aggregates. The authors of the publication [24] developed a unified elastic modulus model of concrete with natural and recycled aggregates, taking into account four input data: dosage, coarse aggregates/cement content, effective water/cement content, and coarse aggregates water absorption. The pioneering unified design equations lead to sustainable and cleaner concrete design.

A factor analysis study [25] revealed that the content of recycled coarse aggregates and the reactivity index of cementitious materials affect the elastic modulus, water absorption of recycled coarse aggregates, and the ratio of aggregates to cementitious materials.

The authors of the publication [26] conducted studies on the stress–strain parameters of concrete made of recycled aggregates reinforced with macro-polypropylene fibers. The addition of fibers has a positive effect on the stress and strain characteristics of concrete. With the increase in the fiber dose, the energy dissipation capacity, peak stress, and peak strain of the samples increase. The increase in strength due to the addition of fibers is higher for RAC samples than for NAC samples. The reduction in peak stress, ultimate strain, strength, and specific strength of concrete samples due to the use of CRA fibers also decreases with the addition of fibers. The negative impact of CRA on concrete properties can be reduced by adding macro-polypropylene fibers.

The results of the research [27] indicate an increase in the peak stress, peak strain, and ultimate strain of concrete samples with the increase in the dose of macro-synthetic fibers. Adding fibers has a better effect on concrete with recycled aggregates compared to concrete with natural aggregates.

Earlier studies by the authors of [28,29,30] and other researchers [31,32,33,34] showed that concrete elements that are formed in a horizontal position are characterized by a fairly large change in compressive strength across their thickness [29].

A certain differentiation of the compressive strength across the thickness of horizontally formed elements is related to the segregation of the components of the concrete mix—aggregate. Recycled aggregate contains, apart from natural aggregate, other components, such as particles of old concrete that have different porosity. Therefore, the question arises as to whether the lighter particles of the recycled aggregate will move upwards more intensively and also to what extent they will affect the differentiation of the compressive strength of the concrete across the thickness of an element. Publications and standards do not show the differences in the compressive strength of samples cut from different layers of cores, which were taken from the elements of a concrete structure made with the addition of recycled aggregate [35,36,37]. Most scientific works emphasize the method of destroying concrete samples that are made of a concrete mix with pure natural aggregate [28,29,30,32,34,38,39,40,41,42,43,44].

The presented premises became the basis for the analysis of the results of tests of the compressive strength of core samples across their thickness. The samples were made with the addition of recycled aggregate and had different sizes. Their strength results were compared with those obtained for concrete elements made of concrete with pure natural aggregate.

## 2. Materials and Methods

For the purpose of testing the compressive strength of concrete across the thickness of a horizontally formed element, recipes for concrete mixes were developed in order to obtain two concretes with different compressive strengths. For each concrete, concrete mixes were prepared with pure natural aggregate with a grain size of 0–2 mm and 0–16 mm, and also with the addition of 50% of recycled aggregate (4–16 mm).

In order to create concretes with two different compressive strengths, the following were used:

- Cement CEM II/B-V 32.5R, 2.95 kg/dm^3^;
- Pure natural aggregate with a grain size of 0–2 mm (river sand), 2.67 kg/dm^3^;
- Pure natural aggregate with a grain size of 0–16 mm (mine gravel), 2.67 kg/dm^3^;
- Recycled aggregate with a grain size of 4–16 mm (crushed standard cubic particles of concrete containing natural aggregate: sand and gravel), 2.40 kg/dm^3^;
- Potable water taken from the municipal water supply, 1.0 kg/dm^3^;
- FK-88 plasticizer was added to the mixing water during the preparation of concrete mix, 1.18 kg/dm^3^.

The mass share of the ingredients is given in Table 1.

### 2.1. Description of the Experiment

In order to test the compressive strength of the concrete across the thickness of a horizontally formed element made with the addition of recycled aggregate, a research project was undertaken in which the following was carried out:

I. The compressive strength of the produced concrete was checked in accordance with EN 12390-3 [45] on standard cubic samples with a side length of 150 mm. The samples were prepared in accordance with EN 12390-1 [46] and then tested according to standard EN 12390-3 [45] on a Toni Technik compression testing machine (with a rate of loading of 0.5 MPa/s).

The concrete with a low compressive strength (20 MPa), which was prepared with pure natural aggregate, had a water–cement ratio (W/C) equal to 0.75—N20. The concrete mix had the consistency class of F5 (580 mm) or S4 (200 mm) according to EN 206 [47], which was determined on the basis of tests using the following methods: slump table according to EN 12350-5 [48], and slump cone according to EN 12350-2 [49]. The concrete with a compressive strength of 40 MPa (N40), which was prepared with pure natural aggregate, had a W/C ratio of 0.5 and a concrete mix consistency of F2 (410 mm) or S1 (40 mm) according to EN 206 [47].

The concrete with a low compressive strength (20 MPa), which was prepared with the addition of 50% recycled aggregate, had a water–cement ratio (W/C) equal to 0.75—R20. The concrete mix had the consistency class of F4 (520 mm) or S3 (150 mm) according to EN 206 [47], which was determined on the basis of tests using the following methods: slump table according to EN 12350-5 [48], and slump cone according to EN 12350-2 [49]. The concrete with a compressive strength of 40 MPa (R40), which was prepared with the addition of 50% recycled aggregate, had a W/C ratio of 0.5 and a concrete mix consistency of F1 (320 mm) or S1 (20 mm) according to EN 206 [47].

The classes of consistency were not the same, which may have had some effect on the ability of the concrete mix’s components to segregate during compaction.

**Figure 1:**
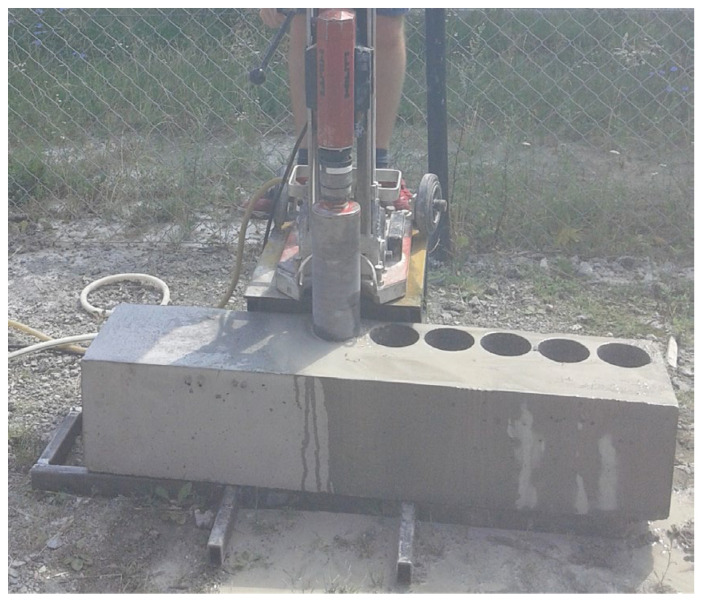
Taking the core samples on a properly prepared stand using a drilling rig with a lace drill.

**Figure 2:**
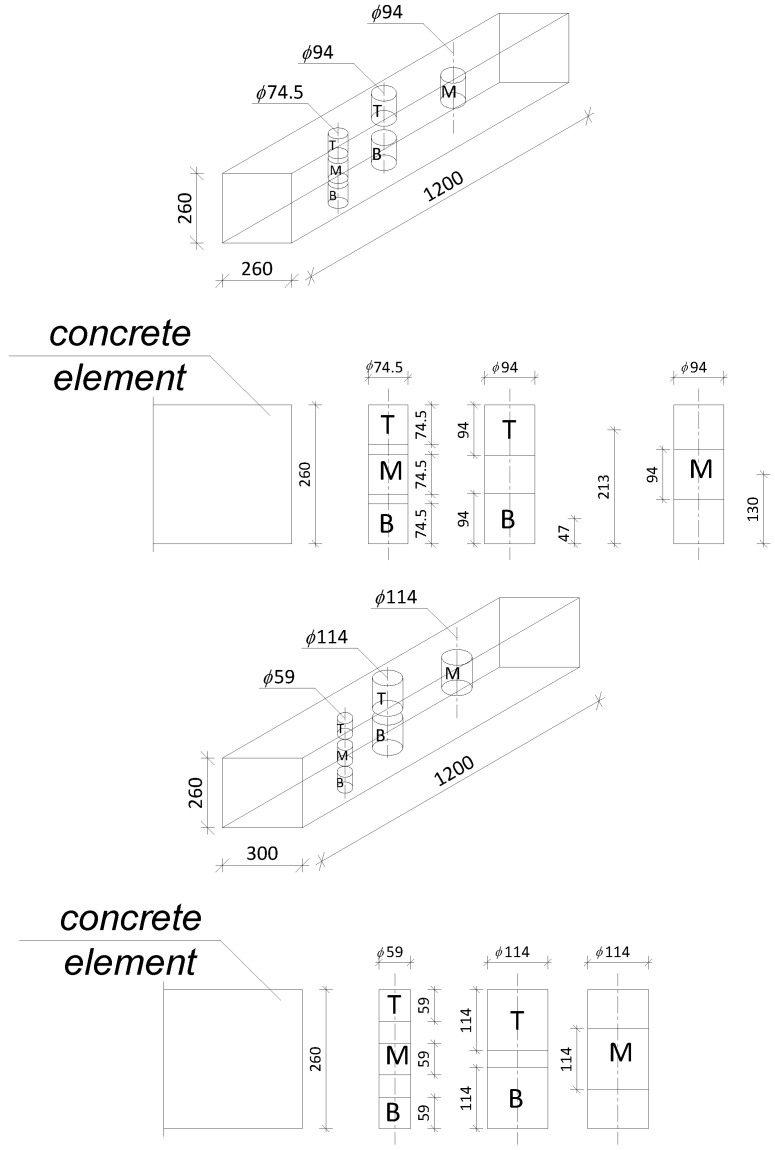
Levels across the thickness of the concrete elements where the compressive strength of concrete was tested: T—top layer; M—middle layer; B—bottom layer.

II. Concrete elements with a thickness of 260 mm were prepared. They were compacted for 5 min with the use of a ϕ 38 mm deep vibrator (with a frequency of 300 Hz).
III. Core samples with a height-to-diameter ratio equal to 1 were cut from cores taken along the direction of concreting (Figure 1) and then tested. Core samples were taken in such a way that half of the sample’s height was placed as low as possible to the bottom layer, the other half was as high as possible to the top layer, and the middle of the sample was halfway up the thickness of the element. Core samples of the diameters 94 mm and 114 mm were taken from two cores to obtain a height-to-diameter ratio equal to 1. Core samples from the top and bottom layers were taken from the first core, and core samples from the middle layer were taken from the second core (Figure 2). According to research [28], the designed compressive strength is for a sample taken in the middle of the height of a concrete cylinder. The concrete elements, after removal from the mold, were stored at a temperature of 20 ± 2 °C and a humidity of 50 ± 15% until the cores were taken. The cores were cut into the core samples. After cutting, the core samples were stored for 30 days at a temperature of 20 ± 2 °C and a humidity of 50 ± 15% until their compressive strength was tested. The core samples were tested 90 days after concreting.

Compressive strength was tested on five levels of the constructed concrete elements, from which six core samples with different diameters were taken. Then, the expanded uncertainty for the mean value of the strength results was calculated. In order to determine the expanded uncertainty for the mean values of the test results, the coverage factor *k* = 2 (at the 95% level) was assumed.

**Figure 3:**
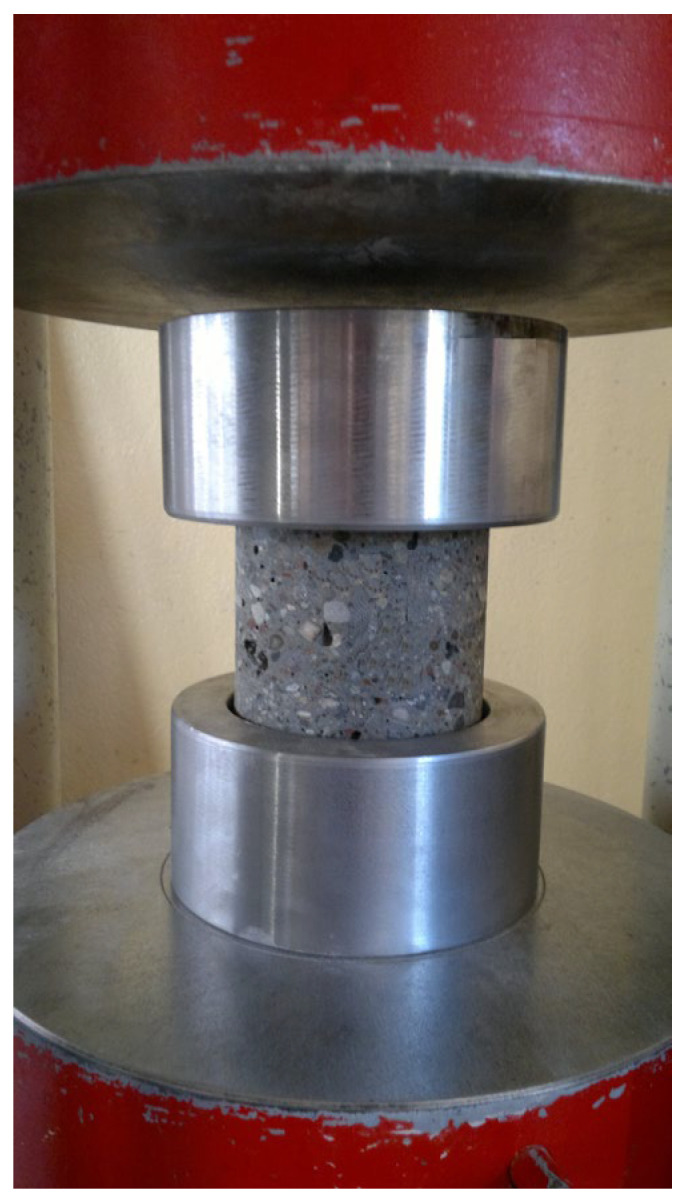
A core sample during the compressive strength test in a testing machine.

To determine the compressive strength of the core samples, steel discs with neoprene pads (Figure 3) according to the ASTM C 1231/C1231M-00 standard [50] were used. The applied research methodology enabled the destructive force during the compressive strength test of the core samples (taken from the top and bottom layers of concrete elements) to be measured. It was not necessary to level the surface of the core samples in accordance with the EN 12504-1 standard [51]. The compressive strength was tested according to standard EN 12390-3 [45] on a DP 1600 compression testing machine (with a rate of loading of 0.5 MPa/s).

### 2.2. Processing of the Research Results

The compressive strength can be calculated using the following equation:

(1) $$f_{c}=\frac{F}{A_{c}}$$

where

*f_c_* is the compressive strength in MPa (N/mm^2^);

*F* is the maximum load at failure in N;

*A_c_* is the cross-sectional area of the specimen (in mm^2^) on which the compressive force acts.

The mean value of the strength results is given by equation:

(2) $$f_{cm}=\frac{1}{n}\sum_{i=1}^{n}f_{c}$$

where

*f_cm_* is the mean value of the compressive strength in MPa (N/mm^2^);

*f_c_* is the value of the compressive strength in MPa (N/mm^2^);

*n* is the number of values.

The expanded uncertainty for the mean value of the strength results is given by equation:

(3) $$U=k\cdot u_{c}$$

(4) $$u_{c}=\sqrt{\frac{1}{n\cdot \left(n-1\right)}\sum_{i=1}^{n}{\left(f_{c-i}-f_{cm}\right)}^{2}}$$

where

*U* is the expanded uncertainty for the mean value of the strength results in MPa (N/mm^2^);

*u_c_* is the standard uncertainty for the mean value of the strength results in MPa (N/mm^2^);

*f_cm_* is the mean value of the compressive strength in MPa (N/mm^2^);

*f_c−i_* is the value of the compressive strength of the i-th sample in MPa (N/mm^2^);

*k* is the coverage factor for the mean values of the test results;

*n* is the number of values.

### 2.3. Statistics

The coverage factor *k* = 2 (consistent with international practice) was used to calculate the expanded uncertainty for the mean values of the test results at the 95% level of confidence. The coverage factor *k* is associated with the interval defined by *U* = *k* · *u_c_*. Normally, *k* is in the range of 2 to 3. For the normal distribution, *u_c_* is reliable for the standard deviation of measurement, *U* = 2 · *u_c_* (*k* = 2, at about the 95% level of confidence), and *U* = 3 · *u_c_* (*k* = 3, at a greater than the 99% level of confidence). Excel was used to perform statistical analysis of the research results.

## 3. Results

### 3.1. Compressive Strength

To analyze the compressive strength of the concrete in the different layers of the concrete element, the values of the average compressive strength were calculated for six core samples (with a diameter of 59 mm, 74.5 mm, 94 mm, and 114 mm) taken from each layer of the element.

The calculated average compressive strengths of the core samples and the expanded uncertainties determined for them in the case of all the concrete series are presented graphically in the four figures below. The numbers 59, 74.5, 94, and 114 on the vertical axes of the graphs represent the diameters of the core samples in millimeters.

**Figure 4:**
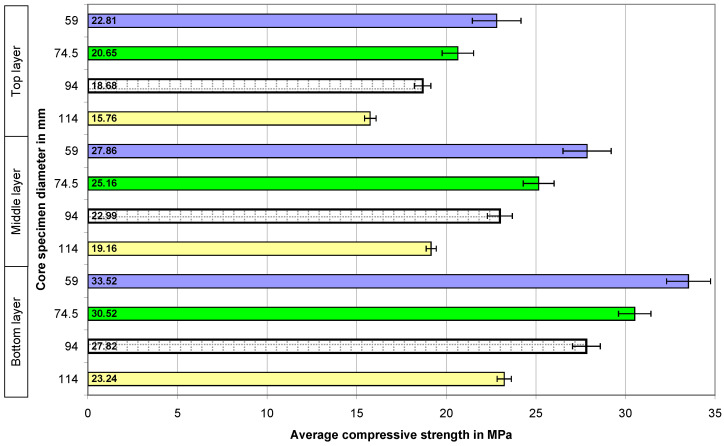
The average compressive strength of the core samples in the case of the 20 MPa concrete with natural aggregate—N20.

Figure 4 shows the results of the compressive strength of the concrete tested at different levels of an element in the case of the 20 MPa concrete series (with pure natural aggregate). The values of the bars in the diagram show the average compressive strength of the concrete determined on the samples with different diameters. The presented results indicate a clear change in the strength of the concrete with regard to the diameter of the core samples. This confirms the existence of the relationship that the increase in strength occurs with a decrease in the diameter of the sample. The change in concrete strength across the height of the element is clearly visible—the top layer is the weakest, and the bottom layer is the strongest.

**Figure 5:**
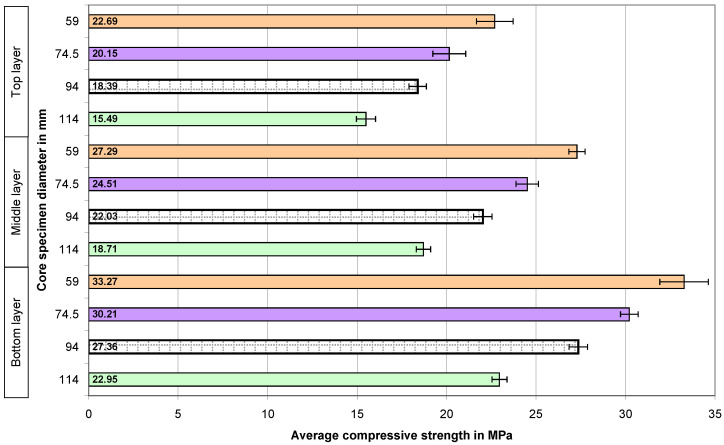
The average compressive strength of the core samples in the case of the 20 MPa concrete with 50% recycled aggregate—R20.

Figure 5 shows the test results for the low-strength concrete (20 MPa) with the addition of recycled aggregate. The change in the compressive strength of the core samples (with diameters of 59 mm, 74.5 mm, 94 mm, and 114 mm) taken from the three layers—bottom, middle, and top—of the concrete element is shown in Figure 5. Replacing some of the aggregates with recycled aggregate did not significantly change the relationship that appeared in the case of the concrete with natural aggregate [28,29,30,32,34,38,39,40,41,42,43,44].

**Figure 6:**
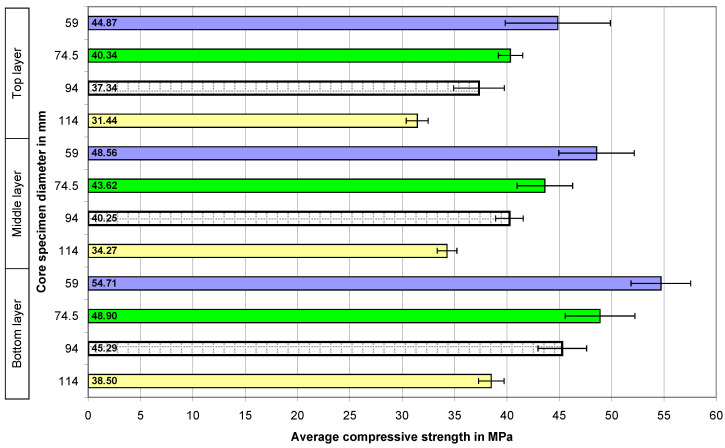
The average compressive strength values of the concrete core samples in the case of the 40 MPa concrete with natural aggregate—N40.

The next tested concrete was the concrete with a strength of 40 MPa (Figure 6). In this case, the previously shown dependencies are also clearly visible—the smaller the sample, the higher the damage strength, and also, the higher the location of concrete in the element, the lower its strength.

**Figure 7:**
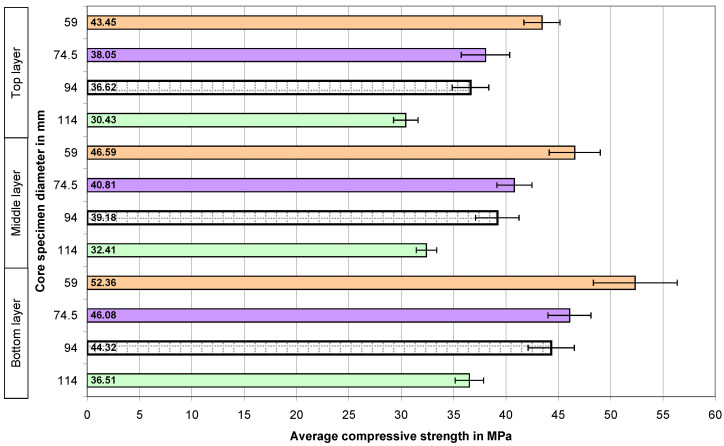
The average compressive strength of the core samples in the case of the 40 MPa concrete with a 50% share of recycled aggregate—R40.

The addition of recycled aggregate did not affect the perceptible change in the relationships (Figure 7), which were emphasized during the initial evaluation of the results of the tests of concrete of the same series (40 MPa) made with natural aggregate (Figure 5).

**Figure 8:**
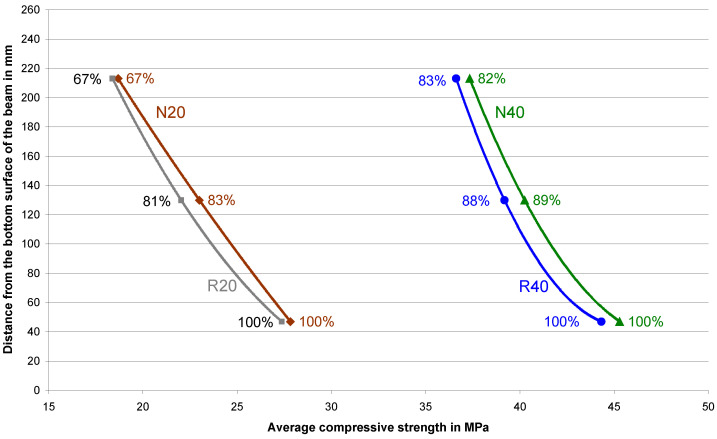
An example of the distribution of the average compressive strength of concrete across the height of the cross-section in the tested concrete element for core samples with a diameter of 94 mm for the series: 20N, 20R, 40N, and 40R.

Figure 8 shows the change in the compressive strength of concrete across the height of the cross-section of an element (260 mm thick), which is expressed as a percentage of the strength of the bottom layer. The top layer is the weakest, and the bottom layer is the strongest, representing 100% of the compressive strength for core samples with a diameter of 94 mm. Due to the fact that the concrete elements from which the core samples with a diameter of 94 mm were taken had a height of 260 mm, the compressive strength of the concrete was tested at the following levels (measured from the bottom): 47 mm (half the height of the core sample with a diameter of 94 mm), 130 mm (center of the element), 213 mm (260 mm minus half the height of the 94 mm diameter core sample).

### 3.2. Compressive Strength Statistics

The expanded uncertainty *U* = *k* · *u_c_* was determined for the obtained mean results of the compressive strength of core samples (Table 2). The expanded uncertainty for the mean values of the test results was determined at the confidence level of 95%, and the coverage factor of *k* = 2 was adopted. *u_c_* is the standard deviation of the measurement.

### 3.3. Conversion Coefficients

The calculated conversion coefficients (Table 3 and Table 4) enabled the compressive strength of concrete to be determined on the core samples (with a diameter of 94 mm) taken from the bottom of the element in the case when the strength was tested on core samples with diameters: 59 mm, 74.5 mm, 114 mm.

It can be noticed (Table 3 and Table 4) that there is a similarity between the compressive strength designated on samples (with different diameters) made of concrete with natural aggregate and samples made of concrete with the addition of recycled aggregate.

**Figure 9:**
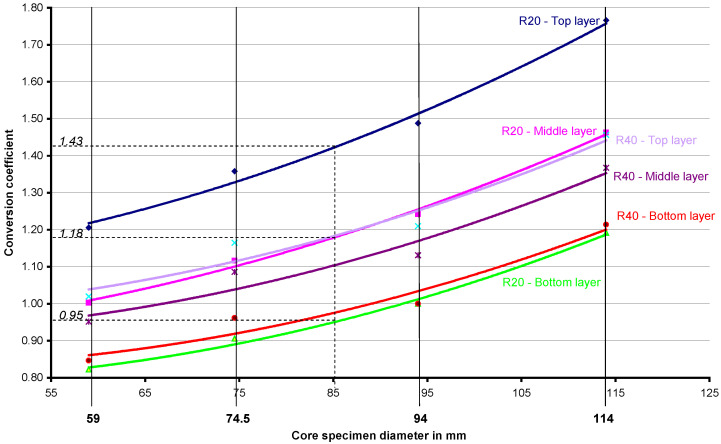
Conversion coefficients that enable the compressive strength of the concrete tested on core samples of any diameter to be converted into the compressive strength of the concrete tested on cubic samples with an edge length of 150 mm. Concrete with the addition of recycled aggregate.

The dependencies that allow the compressive strength of the concrete for core samples of any diameter to be converted into the compressive strength of the concrete for cubic samples with an edge length of 150 mm (standard samples) can be seen to be useful. The compressive strength of the concrete tested on a cube specimen with an edge length of 150 mm corresponds to the compressive strength of a core sample with a diameter of 94 mm (with a height-to-diameter ratio of 1 (Figure 9)).

### 3.4. Recommendation

A core sample with a diameter of 85 mm was taken from a slab that was made of concrete with the addition of recycled aggregate. From this sample, three core specimens with a diameter of 85 mm were cut, and their height-to-diameter ratio was equal to 1. What is the normative compressive strength of concrete when it is of low strength (of about 20 MPa)? The answer (the results for this diameter have not been verified experimentally) is that the strength of the core sample cut from the bottom layer of the slab should be multiplied by 0.95 (Figure 9); when the sample is cut from the middle layer of the slab, its compressive strength should be multiplied by 1.18 (Figure 9), and the compressive strength of the sample taken from the top layer must be multiplied by 1.43 (Figure 9) in order to obtain the normative concrete strength.

For concretes with the addition of recycled aggregate and a compressive strength close to 40 MPa, the conversion factors will have different values: 0.98, 1.11, and 1.19, respectively (Figure 9).

## 4. Discussion

The problem of calculating the compressive strength of core samples of different diameters has been discussed many times in the technical literature [43,44,52,53,54,55] and mainly concerned concrete with natural aggregate. Nowadays, much emphasis is put on concrete with the addition of recycled aggregate; however, there are no published results referring to concrete with such components.

In response to the lack of conversion coefficients in the literature for the values of compressive strength tested on core samples of different diameters, which are taken from concrete with the addition of recycled aggregate, such coefficients were proposed. The proposed coefficients enable the value of the compressive strength of a core sample with a diameter of 94 mm (taken from the bottom of the element) to be calculated in the case of having the values of the compressive strength of core samples of a different diameter (samples taken at a different height of the element).

Standard EN 13791 [35] specifies that “testing a core sample with a length equal to the nominal diameter of 100 mm provides a strength value that corresponds to the strength of a cubic sample with a length of its side equal to 150 mm, which was made and matured under the same conditions”. Among the considered diameters, the diameter of 94 mm is the closest to that recommended by the EN 13791 standard [35]. Therefore, the compressive strength of concrete for samples with different diameters was converted into the value of the strength of samples with this diameter. The factors can be used to test recycled concrete if the compressive strength of the concrete in a structure is close to the strength of the tested elements (20 MPa, 40 MPa).

The differentiation of the strength values of core samples with different diameters, which were taken from the top, middle, and bottom layers of concrete elements made with the addition of natural aggregate and recycled aggregate, is similar. The conducted tests showed that the compressive strength tested on various core samples not only depends on their diameter but also on their strength, which was determined on cubic standard samples. The small diameter of the core sample affects the increase in the compressive strength because of the increased friction force between the neoprene pad and the concrete and prevents destruction. For this reason, the transition from the compressive strength of core samples with different diameters to the strength that would be obtained on cubic samples is a two-parameter dependency.

To test the compressive strength of concrete, recycled aggregate from concrete elements was used. Recycled materials added to concrete are not only building materials [7,8,9]. Therefore, it is necessary to continue testing the compressive strength of core samples taken from concrete with recycled but non-building materials. It is worth considering testing the change in compressive strength over the thickness of high-strength concrete elements [7] with recycled aggregate.

There is contradictory information in the publications on whether adding recycled aggregate to concrete improves the strength [11] or not [4,10]. The obtained research results indicate a very small change in the compressive strength of concrete and a decrease in the consistency class. For the tests, recycled aggregate with a grain size of 4–16 mm (crushed concrete elements) was used.

The authors of publications [14,15,16,17] indicate the influence of the height-to-diameter ratio of the core samples taken on the change in the compressive strength of concrete. The obtained results of the compressive strength tests of core samples were carried out only for the height-to-diameter ratio equal to 1. This assumption excluded another parameter influencing the compressive strength of core samples.

The studies of the authors [28,29,30] and other researchers [31,32,33,34] have shown that concrete elements with natural aggregate formed in a horizontal position are characterized by a relatively large change in compressive strength throughout the thickness. The obtained results of the tests of compressive strength through the thickness of the concrete element with the addition of recycled aggregate also indicate a change in compressive strength.

The obtained test results confirm that the compressive strength of large-diameter concrete core samples is lower than the compressive strength of small-diameter concrete core samples [20]. The results of the research indicate that the size of the core sample affects the compressive strength of concrete and confirm the research results presented in publications [15,16,17,18].

The use of a 59 mm diameter core sample excludes the presence of rebar in the sample, which affects the compressive strength test of concrete in the structure [21]. When taking core samples with fiber-reinforced concrete, it is impossible to avoid fibers that affect the compressive strength [26].

## 5. Conclusions

In the study of the compressive strength of concrete on the thickness of the concrete element, core samples were used. The following conclusions can be drawn from the experimental investigation:

- The compressive strength of the core samples of concrete with pure natural aggregate and with the addition of recycled aggregate from the top layers was about 17% lower (17.1% for the concrete with pure natural aggregate, 17.8% for the concrete with the addition of recycled aggregate) than the strength of the core samples from the bottom layer. This relationship corresponds to the designed compressive strength of the concrete equal to 40 MPa.
- The compressive strength of the core samples of concrete with pure natural aggregate and with the addition of recycled aggregate from the top layers was about 33% lower (33.3% for the concrete with pure natural aggregate, 33.6% for the concrete with the addition of recycled aggregate) than the strength of the core samples from the bottom layer. This relationship corresponds to the designed compressive strength of the concrete equal to 20 MPa.
- When determining the compressive strength of concrete in a structure, the variation in compressive strength across the thickness of the concrete element should be taken into account.
- Thin concrete elements must be carefully formed by the contractors. The contractors must have experience in pouring and compacting concrete mixtures of various structural elements.
- The quality of the concrete made or the technical condition of the building must be determined on the basis of the compressive strength of core samples taken from different layers (there is compression) of horizontally formed concrete elements.
- The conclusion coefficients are held if the height-to-diameter ratio is equal to 1 and if the concrete slab thickness is up to 260 mm.
- The conversion coefficients that enable the compressive strength of the concrete tested on core samples of various dimensions to be converted onto the normative compressive strength after 28 days.

## Figures and Tables

**Table 1:** Concrete mix formulas for the concretes with different compressive strengths.

fcm	Concrete Series	Components of the Concrete Mix	Mass of Components per 1 m^3^ [kg]
20 MPa	N20	Cement	275.0
		Sand	784.7
		Gravel	1083.7
		Water	206.3
	R20	Cement	275.0
		Sand	784.7
		Gravel	541.7
		Recycled aggregate	490.7
		Water	206.3
40 MPa	N40	Cement	300.0
		Sand	636.6
		Gravel	1352.7
		Water	150.0
		Plasticizer	3.0
	R40	Cement	300.0
		Sand	636.2
		Gravel	676.0
		Recycled aggregate	611.2
		Water	150.0
		Plasticizer	3.5

**Table 2:** The expanded uncertainties for the mean value of the strength results in MPa.

Concrete Series	U [MPa]											
	ϕ 59 mm			ϕ 74.5 mm			ϕ 94 mm			ϕ 114 mm		
	Bottom Layer	Middle Layer	Top Layer	Bottom Layer	Middle Layer	Top Layer	Bottom Layer	Middle Layer	Top Layer	Bottom Layer	Middle Layer	Top Layer
**N20**	1.23	1.34	1.36	0.91	0.86	0.88	0.78	0.70	0.46	0.40	0.27	0.32
**R20**	1.36	0.46	1.02	0.50	0.63	0.93	0.51	0.51	0.49	0.42	0.40	0.54
**N40**	2.84	3.60	5.02	3.33	2.64	1.17	2.33	1.31	2.42	1.22	0.94	1.04
**R40**	4.00	2.44	1.73	2.05	1.68	2.31	2.20	2.08	1.75	1.37	0.98	1.17

**Table 3:** Conversion coefficients that enable the compressive strength of the concrete tested on core samples of various dimensions to be converted onto the compressive strength of a core sample (with a diameter of 94 mm) taken from the bottom of the element. Concrete with pure natural aggregate.

	Dimensions in mm	Conversion Coefficient		
		Bottom Layer	Middle Layer	Top Layer
N20	ϕ 59 × 59	0.83	1.00	1.22
	ϕ 74.5 × 74.5	0.91	1.11	1.35
	ϕ 114 × 114	1.20	1.45	1.77
R20	ϕ 59 × 59	0.83	0.93	1.01
	ϕ 74.5 × 74.5	0.93	1.04	1.12
	ϕ 114 × 114	1.18	1.32	1.44

**Table 4:** Conversion coefficients that enable the compressive strength of the concrete tested on core samples of various dimensions to be converted onto the compressive strength of a core sample (with a diameter of 94 mm) taken from the bottom of the element. Concrete with 50% of recycled aggregate.

	Dimensions in mm	Conversion Coefficient		
		Bottom Layer	Middle Layer	Top Layer
N40	ϕ 59 × 59	0.82	1.00	1.21
	ϕ 74.5 × 74.5	0.91	1.12	1.36
	ϕ 114 × 114	1.19	1.46	1.77
R40	ϕ 59 × 59	0.85	0.95	1.02
	ϕ 74.5 × 74.5	0.96	1.09	1.16
	ϕ 114 × 114	1.21	1.37	1.46

## Data Availability

The original contributions presented in this study are included in the article. Further inquiries can be directed to the corresponding author(s).
